# Predicting Fungemia in the ICU: Unveiling the Value of Weekly Fungal Surveillance and Yeast Colonisation Monitoring

**DOI:** 10.3390/jof10100674

**Published:** 2024-09-27

**Authors:** Pedro Suárez-Urquiza, Javier Pemán, Monica Gordon, Patricio Favier, Paula Muñoz-Brell, Jose Luis López-Hontangas, Alba Ruiz-Gaitán

**Affiliations:** 1 Department of Medical Microbiology, University and Polytechnic La Fe Hospital, 46026 Valencia, Spain; javier.peman@gmail.com (J.P.); patriciofavier@hotmail.com (P.F.); paulamb23diciembre@gmail.com (P.M.-B.); lopez_jlu@gva.es (J.L.L.-H.); 2 Severe Infection Research Group, Health Research Institute La Fe, 46026 Valencia, Spain; 3 Department of Intensive Care Unit, University and Polytechnic La Fe Hospital, 46026 Valencia, Spain; mngrsh@hotmail.com

**Keywords:** candidemia, surveillance, colonisation, candidemia predictors, *Candida auris*, intensive care unit

## Abstract

Fungemia remains a major threat in intensive care units (ICUs), with high mortality rates despite advances in diagnostics and treatment. Colonisation by yeasts is an independent risk factor for fungemia; however, its predictive utility requires further research. In this 8-year study, we analysed 38,017 samples from 3206 patients and 171 fungemia episodes as part of a weekly fungal surveillance programme. We evaluated species-specific colonisation patterns, the predictive value of the Colonisation Index (CI) and Corrected Colonisation Index (CCI), and candidemia risks associated with different yeast species and anatomical site colonisation. Our results showed that *C. auris*, *N. glabratus*, and *C. parapsilosis* colonisation increased with longer hospital stays (0.8% to 11.55%, 8.13% to 16.8%, and 1.93% to 5.14%, respectively). The CI and CCI had low discriminatory power (AUROC 67% and 66%). Colonisation by any yeast genera demonstrated high sensitivity (98.32%) and negative predictive value (NPV) (95.90%) but low specificity and positive predictive value (PPV) (23.90% and 6.64%). Tracheal and urine cultures had the highest PPV (15.64% and 12.91%), while inguinal cultures had the highest NPV (98.60%). *C. auris* (12.32%) and *C. parapsilosis* (5.5%) were associated with a higher fungemia risk (log-rank < 0.001). These findings support the use of weekly surveillance to better stratify the fungemia risk and optimise antifungal use in ICUs.

## 1. Introduction

Fungemia represents a significant infectious threat, notably among patients in intensive care units (ICUs), accounting for 15–30% of infections in these units [1,2]. Despite advancements in diagnosis and treatment, the mortality rates associated with fungemia remain high, ranging from 30 to 60% [3,4], with an attributable mortality rate of approximately 30% [5,6]. Early antifungal therapy is crucial, as it reduces the mortality associated with fungemia and its incidence [7,8,9] However, indiscriminate use of antifungals is expensive, can select for resistant species, and increases adverse drug reactions [10].

More than 190 species of *Candida* are known, but 6 species and related genera are predominantly responsible for human infections: *Candida albicans*, *Candida parapsilosis* complex (including *C. parapsilosis* sensu stricto, *C. orthopsilosis*, and *C. metapsilosis*), *Nakaseomyces glabratus* (formerly *Candida glabrata*), *Candida tropicalis*, *Pichia kudriavzevii* (formerly *Candida krusei*), and, in certain regions, the emerging *Candidozyma auris* (formerly *Candida auris*) [11,12,13,14]. *C. albicans* is the most frequent fungal pathogen, but lately, other yeast species represent more than 50% of invasive yeast infection cases [11,15]. In addition, there are important geographical variations in yeast species involved in invasive infections. [16].

Fungemia typically occurs after host colonisation by *Candida* species [17,18,19,20,21]. Understanding the patterns of colonisation may help to develop strategies to prevent candidemia or the horizontal spread of strains such as fluconazole-resistant *C. auris* or *C. parapsilosis*.

Many studies have investigated colonisation as a risk factor for fungemia [17,18,19,20,21]; however, these studies lack differentiation between yeast genera responsible for infections in humans and often limit the number of anatomical locations surveyed. Our study evaluates the colonisation and differentiation of different yeast genera in multiple body sites. This has been facilitated by an ongoing weekly fungal surveillance programme implemented for all ICU patients since 2016 in the context of a *C. auris* outbreak. By differentiating among species, we can evaluate the risk associated with each, improving the applicability of our results to different clinical settings. The aim of this study is to analyse the colonisation patterns of different yeast species over long ICU stays, to assess the efficacy of commonly used predictive scores (the Colonisation Index and Corrected Colonisation Index) [22], and to evaluate the predictive values (specificity, positive predictive value (PPV), and negative predictive value (NPV)) of colonisation by each yeast. By addressing these objectives, we intend to contribute to the risk stratification of fungemia in ICU patients.

## 2. Materials and Methods

### 2.1. Patient Selection Criteria

This retrospective study was conducted from May 2016 to December 2023 at La Fe University Hospital, a 1000-bed tertiary care centre in Valencia, Spain. It included the hospital’s medical and surgical intensive care units, each with 36 beds. Patients over 14 years admitted to these units for at least seven days were studied.

### 2.2. Sample Collection

Samples were collected upon ICU admission and subsequently on a weekly basis following standard procedures. Surveillance sites, which were selected based on clinician criteria, included the throat, axilla, groin, rectum, urine, and tracheal/bronchial regions. Standard sterile culture swabs containing liquid Amies media (Cliniswab™, Canelli, Italy) were used to sample the oropharynx, perineum, axilla, and rectum. Catheter urine and tracheal/bronchial aspirates were collected in sterile tubes.

### 2.3. Fungal Surveillance Cultures

Samples were plated onto CHROMagar *Candida* agar (MAIM, Barcelona, Spain) and CHROMagar *Candida* agar supplemented with 32 mg/mL of fluconazole (MAIM, Barcelona, Spain) using a quantitative urine streaking method. The plates were incubated for 48 h at 37 °C. Colonies were differentiated by colour and growth characteristics, while Matrix-assisted laser Desorption/Ionisation–Time of Flight (MALDI-ToF) (Bruker, Billerica, MA, USA) was used in cases where definitive identification was needed. The growth of each species was quantified from <10 to >1000 colonies. Heavy colonisation of a body site was identified when the colony count was greater than 500 CFU in axillary, oropharyngeal, rectal, or inguinal samples, greater than 50 CFU in tracheal/bronchial samples, or greater than 50,000 CFU/mL in urine samples.

### 2.4. Definitions

Colonisation was defined as the isolation of one of the yeast species studied from at least one body site. The Colonisation Index (CI) was defined as the number of positive cultures to the number of body sites surveyed. The Corrected Colonisation Index (CCI) was calculated as the number of heavily colonised body sites to the number of sites surveyed [22]. Fungemia was identified when a yeast species was isolated in a blood culture.

### 2.5. Statistical Analysis

Surveillance culture results were compared among patients who developed fungemia and those who did not. Comparative analysis was performed using the χ^2^ test and the Fisher test for categorical variables and an unpaired *t*-test or the Mann–Whitney–Wilcoxon test for continuous variables as appropriate. The association between colonisation by different yeast species and the onset of fungemia probability was analysed using Kaplan–Meier survival curves and the log-rank test. Sensitivity, specificity, PPV, and NPV were calculated for each colonisation site, with a CI ≥ 0.5 and a CCI ≥ 0.4 for each species, based on predetermined thresholds [22], choosing the max CI and CCI before yeast bloodstream infection onset. Receiver operating characteristic (ROC) curves were utilised to assess the predictive efficiency of these indices. A multivariable logistic model was used to determine colonisation cases that were independently related to fungemia, adjusting for sex, age, ICU stay length, and surgery. Variables that were strongly correlated with those already in the model were left out of the analysis (i.e., admission diagnosis and surgery). Statistical analysis was performed using R statistics 4.2.3., and a *p*-value < 0.05 was considered significant.

## 3. Results

### 3.1. Patient Characteristics

**Figure 1:**
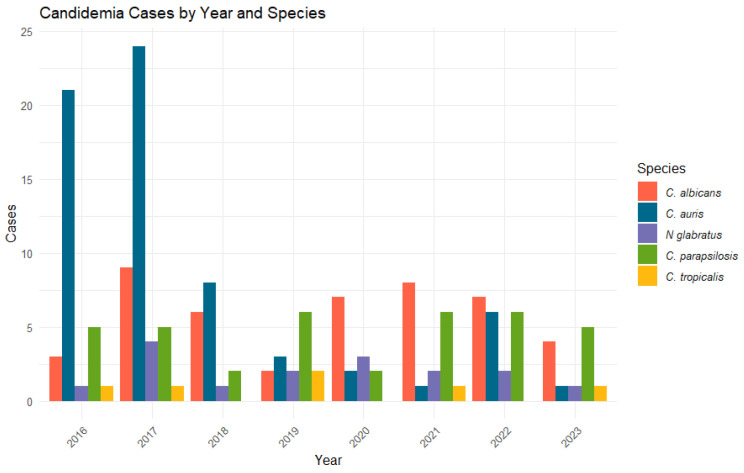
Fungemia cases by yeast species and year.

We analysed 38,017 samples from 3206 patients, including 8919 (23.46%) rectal, 5237 (13.78%) tracheal/bronchial, 5407 (14.22%) perineal, 5450 (14.34%) axillar, 7875 (20.71%) urine, and 5129 (13.49%) oropharyngeal samples. A total of 171 fungemia episodes were identified, which were caused by *C. auris* (66, 38.60%), *C. albicans* (46, 26.90%), *C. parapsilosis* complex (all isolates were *C. parapsilosis* sensu stricto) (37, 21.63%), *N. glabratus* (16, 9.35%), and *C. tropicalis* (6, 3.50%); no cases were attributed to *P. kudriavzevii*. Patient characteristics and diagnoses at ICU admission are summarised in Table 1. Study participants were predominantly male (2072, 64.62%), with a mean age of 58.73 (IQR: 70–50). Patients with fungemia were more likely to be admitted to the surgical ICU, with all surgical diagnoses linked to a higher rate of fungemia. Mortality was higher among patients with fungemia (48.48% vs. 29.72%, *p* < 0.001). Between 2016 and 2018, *C. auris* was the predominant species in fungemia cases, but from 2019 to 2023, there was a more balanced distribution among fungemia-causing species (Figure 1).

### 3.2. Colonisation Patterns of Yeast Species

**Figure 2:**
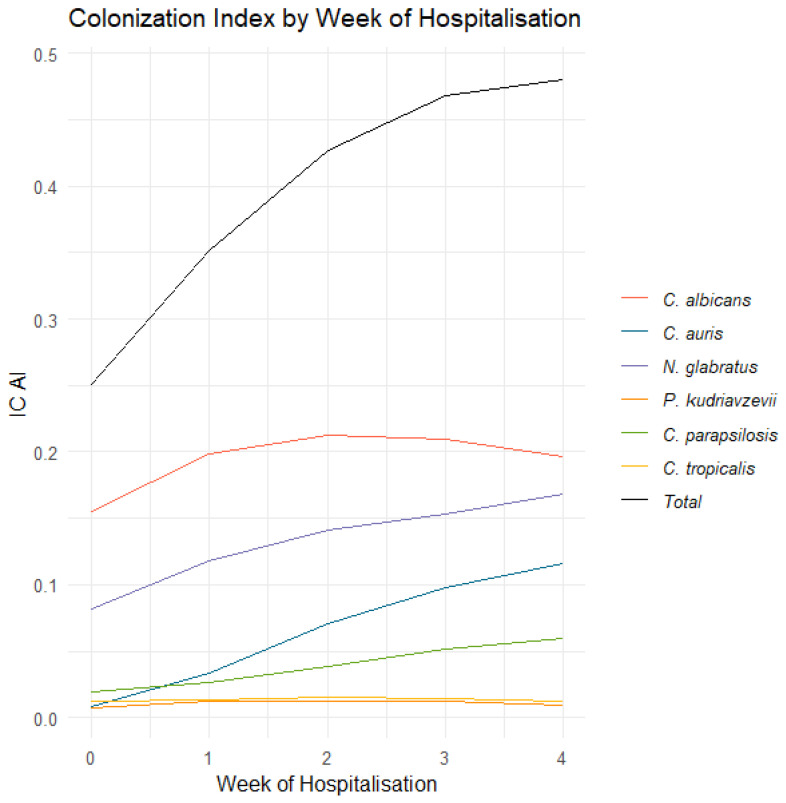
Colonisation Index by week of hospitalisation.

At ICU admission, 25.09% of patients were colonised by any yeast species. These included *C. albicans* (15.5%), *N. glabratus* (8.13%), *C. parapsilosis* (1.93%), *C. auris* (0.8%), *C. tropicalis* (1.2%), and *P. kudriavzevii* (0.7%). By the fourth week of admission, the proportion of patients colonised by yeasts increased to 48.00%. *C. auris*, *C. parapsilosis*, and *N. glabratus* showed a positive trend with increasing length of hospital stay. The colonisation rates for these species by week four rose to 16.8%, 5.14%, and 11.55%, respectively. In contrast, the colonisation rates of *C. albicans*, *C. tropicalis*, and *P. kudriavzevii* remained relatively stable over time, with rates at week four of 19.67%, 1.2%, and 0.9%, respectively (Figure 2).

### 3.3. Fungemia Predictive Values for Colonisation by Each Yeast Species 

Out of all yeast bloodstream infection cases, 28 (16.37%) occurred without prior colonisation by the same yeast species that caused the infection. However, in 19/28 of these cases (67.85%), there was colonisation by a different yeast species before the onset of fungemia. Among these 28 patients, 26 (92.85%) had a recent surgical history, with 14 (50%) undergoing procedures in the week preceding fungemia onset.

The median interval between colonisation and the onset of fungemia was 10 days (IQR: 7–18), and from ICU admission to fungemia onset, the median interval was 18 days (IQR, 10.5–30). For *C. albicans*, these periods were 12 (IQR, 7–21.5) and 14 days (IQR, 9–25), respectively; for *C. auris*, 10 (IQR, 4–18) and 24 (IQR, 16–35) days; for *C. parapsilosis*, 10 (IQR, 9–16) and 19 (IQR, 10–28) days; for *N. glabratus*, 7 (IQR, 4–10) and 16 (IQR, 7–18.5) days; and for *C. tropicalis*, 10 (IQR, 8–29.5) and 12 (IQR, 10–31) days. We analysed predictive values for yeast species’ colonisation (Table 2). All yeast species’ colonisation had an NPV above 98%. The PPVs for all the studied species’ colonisation were below 13%, with *C. auris* having the highest PPV (12.33%), followed by *C. parapsilosis* (5.50%). Implementing a CI ≥ 0.5 resulted in decreased sensitivity but increased specificity and PPV for all species. A similar trend was observed when using a CCI ≥ 0.4 (Table 2).

**Figure 3:**
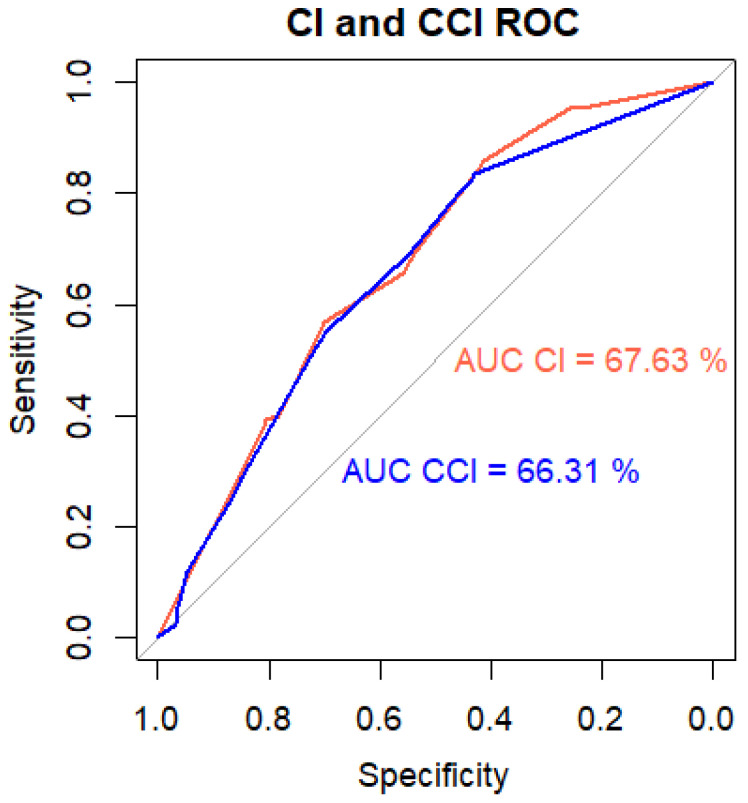
ROC curves for CI and CCI.

ROC analysis of CIs and CCIs revealed areas under the curve (AUCs) of 0.676 [0.64–7.13] and 0.663 [0.624–0.701], respectively (Figure 3). For our population, optimal classification thresholds were 0.36 for the CI and 0.2 for the CCI, with sensitivity and specificity values of 0.86 and 0.41 and 0.836 and 0.43, respectively (Figure 3).

### 3.4. Anatomical Site Preferences of Yeast Species

**Figure 4:**
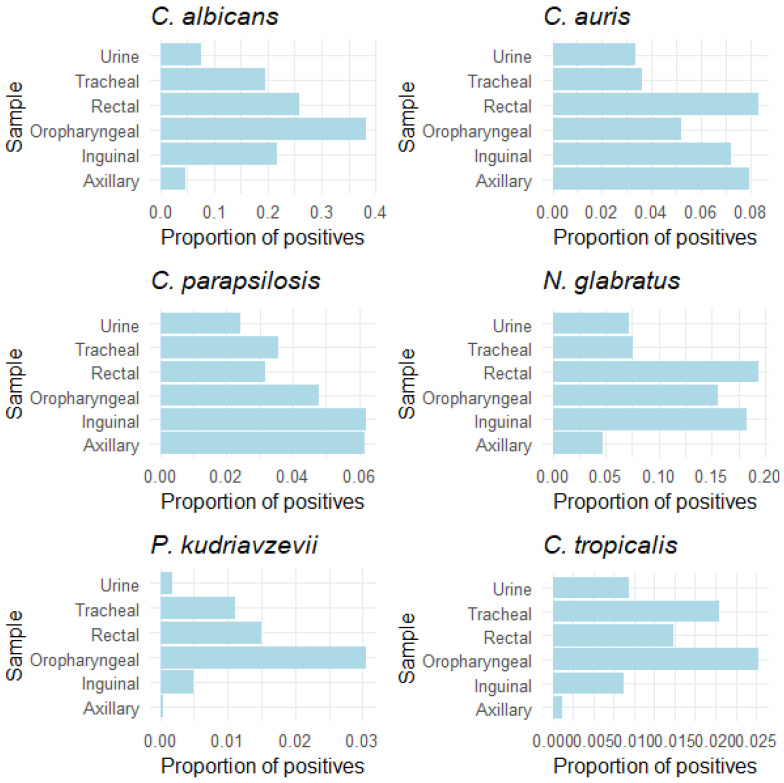
Anatomical preferences for colonisation by each yeast species.

Considering the proportion of cultures positive for each of the yeasts studied, *C. auris* and *N. glabratus* predominantly colonised the rectum, whereas *C. albicans*, *P. kudriavzevii*, and *C. tropicalis* more commonly colonised the oropharynx, and *C. parapsilosis* primarily colonised the inguinal region (Figure 4).

Tracheal/bronchial colonisation by *C. auris*, *C. albicans*, or *C. tropicalis* showed the highest PPVs for predicting subsequent fungemia caused by the same species (32.84%, 7.14%, and 7.14%, respectively). *C. parapsilosis* was most predictive with urine colonisation, and *N. glabratus* was most predictive with colonisation in the oropharynx. Accounting for colonisation and fungemia by all yeast species, the incidence of fungemia was highest with tracheal colonisation (15.64%), followed by urine colonisation (12.91%). Inguinal and axillar cultures demonstrated the highest NPVs among the places studied (98.60% and 97.53%, respectively). However, distinct yeast species demonstrated varying values across colonisation sites (Table 3).

### 3.5. Yeast Colonisation Risk

**Figure 5:**
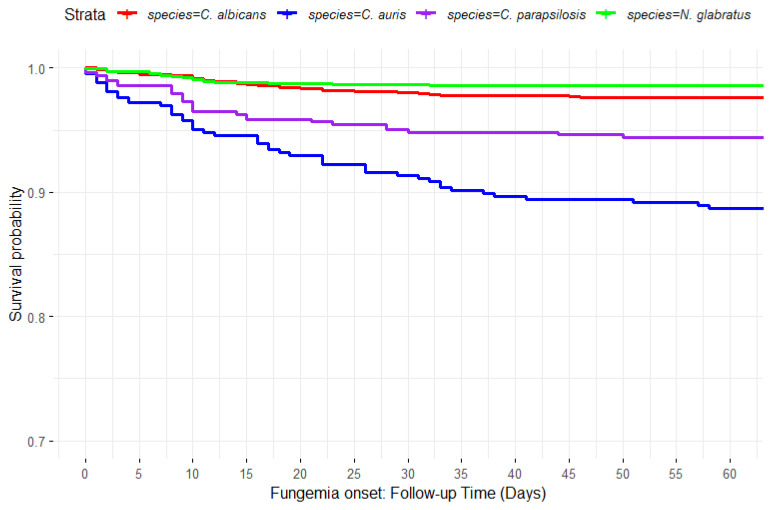
Fungemia probability by yeast species’ colonisation.

The univariate analysis revealed a significant association between colonisation status and the subsequent development of fungemia for every species. Invasive infections occurred more frequently in patients colonised by *C. auris* (53/430, 12.32%) and *C. parapsilosis* (26/472, 5.50%) compared to *C. albicans* (43/1680, 2.55%), *N. glabratus* (16/1180, 1.35%), and *C. tropicalis* (3/98, 3.06%). Kaplan–Meier survival estimates substantiated the increased probability of fungemia in patients colonised with *C. auris* and *C. parapsilosis* (Figure 5, log-rank test *p*-value < 0.001). The significance persisted after the exclusion of *C. auris* from the analysis (log-rank test *p*-value < 0.001). Principio del formularioIn the logistic regression analysis, after controlling for age, gender, length of stay in an ICU, and surgical history, the number of anatomical places colonised by *C. auris* (aOR: 2.34 (2.02–2.70), *p* < 0.001), *C. parapsilosis* (aOR: 2.04 (1.67–2.49), *p* < 0.001), and *C. albicans* (aOR: 1.40 (1.22–1.60), *p* < 0.001) was significantly associated with fungemia caused by those species. Previous surgery was also a significant predictor of fungemia (aOR: 3.82 (2.45–5.95), *p* < 0.001).

## 4. Discussion

Our study reveals distinct colonisation patterns among yeast species, marked by increased colonisation rates of *C. parapsilosis*, *N. glabratus*, and *C. auris* during ICU stays. Additionally, we observed species-specific anatomical preferences correlating with the highest incidence of fungemia, specifically for tracheal and urine colonisation. Surveillance cultures showed low PPVs but high NPVs, emphasising their role in identifying patients at a decreased risk for fungemia. This study also highlights the importance of *C. auris* and *C. parapsilosis* colonisation in the pathogenesis of fungemia.

Contrary to previous studies [18,21,22,23,24], *C. albicans* was not the primary infectious species, although it was the most frequent coloniser. *C. auris* was responsible for 36% of the infections, followed by *C. albicans* (26.90%) and *C. parapsilosis* (21.63%). This shift is probably attributed to this study’s timeframe beginning in 2016, concurrent with the *C. auris* outbreak in our hospital. Notably, the proportion of *C. parapsilosis* fungemia was higher than that reported in the studies previously mentioned.

Our study emphasises the increase in colonisation with increasing hospital stays, with rates nearly doubling from 25% at admission to 48% by the fourth week, consistent with prior research [18]. This underlines the importance of continuous fungal surveillance screening in long-stay ICU patients. *C. auris*, *C. parapsilosis*, and *N. glabratus* demonstrated marked increases over the duration of hospitalisation. This shift in yeast colonisation patterns over time, combined with the distinctive resistance profiles of *C. auris* and *N. glabratus*, could have implications for the implementation of antifungal management strategies in patients with long stays in critical care units. This is supported by a larger interval between ICU admission and fungemia onset for these species, with 14 days for *C. albicans* and 16 and 24 days for *N. glabratus* and *C. auris*, respectively.

As in other studies [18,20], we found high NPVs for all yeast species’ colonisation but low PPVs (6.64%), in line with previous reports [23,24], due to the low incidence of fungemia. The evaluated indices (CI and CCI) showed modest AUCs (67% and 66%, respectively), with high sensitivity and NPVs but low specificity and PPVs for the original breakpoints of 0.5 and 0.4 [22]. This low discriminatory power of the CI and CCI aligns with the outcomes of other external validation studies [21,25,26]. Using colonisation-based indices alone is insufficient for predicting fungemia, and these parameters must be evaluated in conjunction with clinical variables. Consistent with previous research [18,27], few fungemia cases (4.6%, n = 8) appeared without preceding yeast colonisation. A minority of fungemia cases (n = 28, 16.37%) occurred in patients not colonised by the causative yeast species.

Surgical contamination by these species is a plausible factor, as most cases were observed in patients who underwent recent surgery (92.85%). Another hypothesis is the inhibition of the detection of the causative species due to overgrowth by other species; in 67.85% of cases, a different yeast species was present in cultures before fungemia onset.

Tracheal and urine colonisation by yeast species exhibit the highest PPVs. Given that tracheal sampling is more commonly conducted in ventilated and critically ill patients, an elevated rate of fungemia in this group is expected, which may introduce bias in fungemia’s PPVs. Notably, the PPVs for urine colonisation, especially by *C. auris* and *C. parapsilosis*, are significantly high, at 25.44% and 18.84%, respectively. While current guidelines from both the European (ESCMID) and American (IDSA) infectious diseases societies recommend against treating funguria [28,29] except in specific circumstances, the high PPVs associated with these two species might support early antifungal therapy, particularly in those admitted in surgical critical units. While previous studies have identified elevated PPVs for urine colonisation by yeast species [18,30], they did not discriminate between species, overlooking the differences observed in our study. Specifically, the risk associated with urine colonisation by *C. albicans*, *N. glabratus*, *C. tropicalis* is comparatively lower (4.04%, 2.76%, and 5.41%, respectively). Overall, the samples yielding the highest negative predictive values (NPVs) for fungemia were inguinal (98.60%) and axillary (97.53%) samples, but most locations showed high NPVs. This aligns with findings from other studies [18,19,20,24,31] reinforcing the idea that patients with negative fungal surveillance cultures are unlikely to benefit from antifungal treatments.

The Kaplan–Meier survival analysis revealed a different risk of fungemia associated with yeast species’ colonisation, with higher fungemia rates for *C. auris* and *C. parapsilosis* colonisation (12.32% and 5.5%, respectively), as confirmed by a significant log-rank test. This significance persisted after removing *C. auris* from the analysis, making these findings applicable to other regions where *C. auris* is not endemic. While previous research [32,33] has underscored the high rates of fungemia associated with *C. auris* colonisation, comparable to our observed rate (12.32%), these studies did not perform a comparative risk analysis across different yeast species, overlooking the relative colonisation risk presented by each species. The multivariable analysis supports this statement, as colonisation by these species was associated with a blood infection after adjusting for confounding factors.

The retrospective nature and single-centre scope of our study may limit its generalisability. Nonetheless, the analysis of a large number of samples and patients provides robust statistical insights from a substantial dataset. Also, our study began in 2016 with a *C. auris* outbreak in our hospital. While the extended study duration aimed to mitigate the impact of this outbreak on the general fungemia epidemiology, it is possible that the early years particularly influenced the observed results. Additionally, although sample collection is performed in every patient in the ICU, the anatomical site of selection is decided by clinicians, introducing variability in patient comparisons.

Our results suggest that an active fungal surveillance programme could improve clinical practice by identifying patients at risk for fungemia. Specifically, patients with negative cultures may avoid unnecessary antifungal treatment, whereas those with urinary or tracheal colonisation, especially by *C. auris* and *C. parapsilosis*, could be considered at an increased risk for fungemia.

Further multicentre research is essential to validate our findings across diverse healthcare settings. Additionally, targeted studies focusing on the treatment of funguria, particularly caused by *C. auris* and *C. parapsilosis* in critically ill patients, are necessary.

## Figures and Tables

**Table 1:** Patient characteristics.

Variable *n* (%)	Fungemia (*n* = 171)	No Fungemia (*n* = 3035)	OR	*p*-Value
Male sex	122 (71.34%)	1950 (64.25%)	1.38 [0.99–1.95]	0.059
Age ≥ 65	57 (33.33%)	1124 (37.03%)	0.85 [0.61–1.17]	0.32
Surgical ICU admission	111 (64.9%)	1275 (42.0%)	2.54 [1.85–3.53]	<0.001
No positive previous colonisation cultures	8 (4.68%)	1297 (42.73%)	0.06 [0.02–0.11]	<0.001
1–2 previous colonisation cultures	96 (56.14%)	1394 (45.93%)	1.51 [1.11–2.06]	0.004
>2 previous colonisation cultures	68 (39.76%)	344 (11.33%)	5.16 [3.71–7.14]	<0.001
Mortality	80 (48.48%)	904 (29.72%)	2.22 [1.62–3.05]	<0.001
Days of admission (mean ± SD)	25.42 ± 18.58	22.54 ± 13.34	-	0.047
Reason for admission				
Heart surgery	35 (20.47%)	253 (8.34%)	2.88 [1.88–4.16]	<0.001
Polytrauma	26 (15.2%)	193 (6.36%)	2.65 [1.66–4.06]	<0.001
Respiratory infection	20 (11.7%)	620 (20.43%)	0.51 [0.31–0.81]	0.005
Abdominal surgery	15 (8.77%)	126 (4.15%)	2.23 [1.22–3.80]	0.004
Cardiovascular disease	15 (8.77%)	562 (18.52%)	0.42 [0.23–0.70]	0.001
Neurosurgery	14 (8.19%)	78 (2.57%)	3.41 [1.80–5.98]	<0.001
Abdominal infection	10 (5.85%)	120 (3.95%)	1.52 [0.73–2.83]	0.22
Neoplasia	10 (5.85%)	110 (3.62%)	1.67 [0.80–3.11]	0.136
Neurologic disease	8 (4.68%)	503 (16.57%)	0.25 [0.11–0.48]	<0.001
Septic shock	5 (2.92%)	102 (3.36%)	0.89 [0.30–2.01]	0.756
Solid organ transplantation	4 (2.34%)	90 (2.97%)	0.81 [0.24–1.97]	0.81
Haematologic disease	2 (1.17%)	90 (2.97%)	0.41 [0.06–1.32]	0.236
Renal disease	0 (0%)	40 (1.32%)	0 [0.00–1.71]	0.272
Other	7 (4.09%)	148 (4.88%)	0.85 [0.35–1.71]	0.64

**Table 2:** Fungemia predictive values for colonisation by each yeast species.

	*p*	OR	S	E	PPV	NPV
Colonisation						
*C. auris*	<0.001	29.63 [40.57–21.64]	80.30%	87.91%	12.33%	99.53%
*C. albicans*	<0.001	13.13 [23.89–7.22]	93.48%	47.82%	2.56%	99.80%
*C. parapsilosis*	<0.001	14.31 [20.58–9.95]	70.27%	85.82%	5.51%	99.59%
*N. glabratus*	<0.001	-	100.00%	63.25%	1.36%	100.00%
*C. tropicalis*	<0.001	83.74 [251.11–27.93]	83.33%	94.37%	2.72%	99.97%
Any yeast species	<0.001	6.4 [9.22–4.44]	95.32%	23.90%	6.64%	98.90%
IC ≥ 0.5						
*C. auris*	<0.001	24.86 [32.49–19.02]	65.15%	93.01%	16.48%	99.21%
*C. albicans*	<0.001	5.54 [7.76–3.95]	73.91%	66.15%	3.10%	99.43%
*C. parapsilosis*	<0.001	12.72 [17.96–9.02]	40.54%	94.91%	8.57%	99.27%
*N. glabratus*	<0.001	11.73 [20.93–6.57]	75.00%	79.63%	1.83%	99.84%
*C. tropicalis*	<0.001	18.87 [45.19–7.88]	33.33%	97.42%	2.38%	99.87%
Any yeast species	<0.001	3.94 [4.88–3.19]	84.21%	42.50%	7.68%	97.93%
ICC ≥ 0.4						
*C. auris*	<0.001	13.39 [17.84–10.05]	30.30%	96.86%	16.95%	98.50%
*C. albicans*	<0.001	3.42 [4.65–2.52]	39.13%	84.19%	3.50%	98.95%
*C. parapsilosis*	<0.001	18.76 [28.19–12.48]	24.32%	98.32%	14.52%	99.10%
*N. glabratus*	<0.001	21.42 [36.86–12.45]	68.75%	90.69%	3.59%	99.83%
*C. tropicalis*	<0.001	58.33 [141.67–24.02]	33.33%	99.15%	6.90%	99.87%
Any yeast species	<0.001	2.81 [3.29–2.39]	54.97%	69.69%	9.33%	96.46%

**Table 3:** Anatomical sites of yeast species’ colonisation and predictive values for fungemia.

Yeast Species	Sample	*p*	OR	E	S	NPV	VPP
Total							
	Urine	<0.001	3.3 [3.91–2.79]	76.04%	50.98%	95.70%	12.91%
	Tracheal/Bronchial	<0.001	2.46 [2.04–2.96]	58.01%	64.03%	92.99%	15.64%
	Rectal	<0.001	2.68 [2.17–3.32]	36.07%	82.61%	96.41%	9.07%
	Oropharyngeal	<0.001	2.43 [1.87–3.15]	28.99%	85.60%	95.81%	9.59%
	Axillary	<0.001	2.85 [2.17–3.72]	75.30%	48.28%	97.53%	6.71%
	Inguinal	<0.001	4.04 [2.88–5.68]	49.71%	80.36%	98.60%	5.43%
*C. auris*							
	Urine	<0.001	21.88 [16.6–28.83]	96.25%	46.03%	98.46%	25.44%
	Tracheal/Bronchial	<0.001	15.2 [11.15–20.7]	96.23%	37.29%	96.88%	32.84%
	Rectal	<0.001	18.91 [14.27–25.04]	88.65%	70.77%	99.02%	15.75%
	Oropharyngeal	<0.001	9.42 [7.01–12.66]	94.32%	36.21%	97.41%	20.00%
	Axillary	<0.001	-	94.55%	100.00%	100.00%	11.11%
	Inguinal	<0.001	-	94.55%	100.00%	100.00%	10.31%
*C. albicans*							
	Urine	0.0143	2.41 [1.67–3.5]	70.70%	50.00%	98.28%	4.04%
	Tracheal/Bronchial	<0.001	7.49 [5.42–10.35]	89.22%	47.50%	98.98%	7.14%
	Rectal	0.001	2.84 [2.04–3.97]	60.49%	65.00%	98.95%	2.92%
	Oropharyngeal	<0.001	12.6 [6.03–26.32]	50.20%	92.59%	99.74%	3.23%
	Axillary	0.066	2.72 [1.54–4.79]	91.58%	20.00%	98.92%	2.88%
	Inguinal	<0.001	5.19 [3.16–8.52]	70.54%	68.42%	99.47%	2.71%
*C. parapsilosis*							
	Urine	<0.001	14.14 [9.62–20.78]	95.38%	40.63%	98.38%	18.84%
	Tracheal/Bronchial	<0.001	14.27 [9.42–21.63]	97.21%	29.03%	99.02%	12.33%
	Rectal	<0.001	10.66 [7.42–15.32]	94.75%	37.14%	98.95%	10.16%
	Oropharyngeal	<0.001	13.06 [8.71–19.57]	92.89%	50.00%	99.08%	10.83%
	Axillary	<0.001	5.7 [3.3–9.86]	92.61%	31.25%	99.27%	4.03%
	Inguinal	<0.001	10.54 [6.34–17.51]	91.33%	50.00%	99.45%	5.48%
*N. glabratus*							
	Urine	0.048	3.08 [1.7–5.61]	88.52%	28.57%	99.09%	2.76%
	Tracheal/Bronchial	0.150	2.49 [1.29–4.79]	90.12%	21.43%	99.47%	1.30%
	Rectal	<0.001	16.94 [7.94–36.13]	70.76%	87.50%	99.87%	2.12%
	Oropharyngeal	<0.001	38.86 [13.65–110.65]	77.94%	91.67%	99.92%	3.19%
	Axillary	0.006	5.91 [2.83–12.33]	90.79%	37.50%	99.66%	1.97%
	Inguinal	<0.001	21.68 [7.43–63.25]	75.59%	87.50%	99.92%	1.76%
*C. tropicalis*							
	Urine	<0.001	43.49 [17.18–110.06]	98.49%	40.00%	99.87%	5.41%
	Tracheal/Bronchial	<0.001	91.77 [28.59–294.53]	96.83%	75.00%	99.92%	7.14%
	Rectal	0.691	-	96.93%	0.00%	99.77%	0.00%
	Oropharyngeal	<0.001	-	95.27%	100.00%	100.00%	2.70%
	Axillary	0.916	-	99.63%	0.00%	99.81%	0.00%
	Inguinal	0.814	-	98.19%	0.00%	99.81%	0.00%

## Data Availability

The data presented in this study are available on request from the corresponding author.
